# Value of MR and CT Imaging for Assessment of Internal Carotid Artery Encasement in Head and Neck Squamous Cell Carcinoma

**DOI:** 10.1155/2013/968758

**Published:** 2013-01-29

**Authors:** W. L. Lodder, C. A. H. Lange, H. J. Teertstra, F. A. Pameijer, M. W. M. van den Brekel, A. J. M. Balm

**Affiliations:** ^1^Department of Head & Neck Oncology and Surgery, The Netherlands Cancer Institute-Antoni van Leeuwenhoek Hospital, Amsterdam, The Netherlands; ^2^Department of Otorhinolaryngology/Head and Neck Surgery, The University Medical Center Groningen, University of Groningen, 9700 RB Groningen, The Netherlands; ^3^Department of Radiology, The Netherlands Cancer Institute-Antoni van Leeuwenhoek Hospital, Amsterdam, The Netherlands; ^4^Department of Radiology, University Medical Centre Utrecht, Utrecht, The Netherlands; ^5^Department of Otorhinolaryngology, Academic Medical Centre, University of Amsterdam, Amsterdam, The Netherlands; ^6^Institute of Phonetic Sciences, ACLC, University of Amsterdam, Amsterdam, The Netherlands

## Abstract

*Objective*. This study was conducted to assess the value of CT and MR imaging in the preoperative evaluation of ICA encasement. *Methods*. Based upon three patient groups this study was performed. Retrospective analysis of 260 neck dissection reports from 2001 to 2010 was performed to determine unexpected peroperative-diagnosed encasement. Two experienced head and neck radiologists reviewed 12 scans for encasement. *Results*. In four out of 260 (1.5%) patients undergoing neck dissection, preoperative imaging was false negative as there was peroperative encasement of the ICA. Of 380 patients undergoing preoperative imaging, the radiologist reported encasement of the ICA in 25 cases. In 342 cases no encasement was described, 125 of these underwent neck dissection, and 2 had encasement peroperatively. The interobserver variation kappa varied from 0.273 to 1 for the different characteristics studied. *Conclusion*. These retrospectively studied cohorts demonstrate that preoperative assessment of encasement of the ICA using MRI and/or CT was of value in evaluation of ICA encasement and therefore contributively in selecting operable patients (without ICA encasement), since in only 1.5% encasement was missed. However, observer variation affects the reliability of this feature.

## 1. Introduction

Preoperative diagnosis of internal carotid artery (ICA) involvement changes the primary treatment of head and neck tumors. Literature data on carotid encasement in head and neck cancer are scarce. One series reported on a 5% to 10% incidence of cervical lymph node metastases invading the ICA not diagnosed on preoperative imaging using 5 different imaging signs [1]. Encasement of the ICA is both a poor prognostic indicator and often a contraindication to surgical treatment [2]. Removal of lymph node metastases from the ICA may lead to stroke and carotid rupture in 3.3% and 5.5%, respectively [3]. The risk for cerebral damage after removal of the ICA is 3.3% to 30% [1]. Although grafting of the carotid artery, as generally performed in vascular disease and glomus tumors, is possible, it is generally not advocated because the outcome in oncologic patients is dismal [4]. 

Many attempts have been undertaken to classify carotid invasion on preoperative imaging including ultrasound, followed by magnetic resonance imaging (MRI) and computed tomography (CT) scan [1, 2, 5–13].

In 1995 Yousem et al. [2] demonstrated in a series of 49 patients undergoing neck dissection for head and neck tumors clinically suspicious for encasement that more than 270 degrees of circumferential involvement of the ICA on MRI predicted unresectable disease. They reached sensitivity and specificity of 100% and 88%. Assessment of carotid invasion by ultrasonography had sensitivity up to 100% [10–13]. However, in this study we focused on the value of MR and CT imaging. 

Until now, no consensus has been reached on standardization of imaging criteria for defining encasement of the carotid artery. MRI seems to be the most sensitive imaging modality to visualize contrasts between soft tissues structures and therefore should be optimal for the assessment of carotid encasement. Apart from the publications of Pons et al. [1] and Yousem et al. [2], no other studies were performed for classifying carotid encasement on MR imaging. Carotid encasement has a low incidence, but a high impact on treatment planning. This study was conducted to assess the value of CT and MR imaging in the preoperative evaluation of ICA encasement. Therefore we studied 3 patient groups/cohorts retrospectively to review the number of cases with peroperative encasement of the ICA in our institution (group 1) and to assess the prevalence of preoperatively diagnosed encasement of the ICA on CT and MR scans (group 2) and interobserver variation (group 3).

## 2. Materials and Methods

### 2.1. Ethical Considerations

Institutional approval for the study was received. As patient anonymity was preserved patient consent was not required for the retrospective review of records and images.

The results of this study will be presented based upon following three different patient groups.

**Figure 1:**
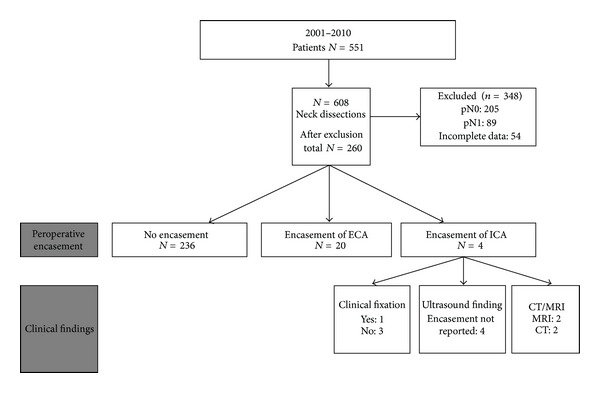
Neck dissections performed between 2001 and 2010. ECA: external carotid artery. ICA: internal carotid artery. This figure shows 551 patients in which 608 neck dissections were performed. In total 260 cases were studied after exclusion. In 236 cases no encasement was found during operation. In 20 cases (7.7%) encasement of the external carotid artery was seen. In four cases encasement of the internal carotid artery was present (4/260 = 1.5%). Two cases had MRI and 2 had CT preoperatively.

*(1) Peroperative Assessment of Encasement of the ICA*. Between 2001 and 2011 a total of 551 patients (608 neck dissections) who had undergone neck dissection in our institution for head and neck squamous cell carcinoma following a pre-surgical MRI or CT workup were selected from our operation database. In our center, patients with a tumor located above the level of the hyoid bone or with an unknown primary tumor are preferentially studied with MR imaging. After a first evaluation of the 608 operation reports, 348 patients were excluded (incomplete data, pathological N0-stage, or pathological N1-stage). Two hundred and sixty operation reports were evaluated for the presence of peroperative carotid encasement (Figure 1). All patients received a (modified) radical neck dissection or salvage selective neck dissection or superselective lymph node dissection after chemoradiation therapy and underwent preoperative evaluation with CT or MR imaging.

**Figure 2:**
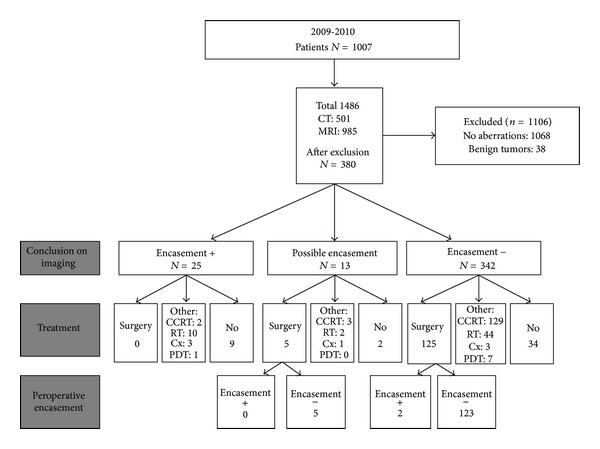
Retrospective analysis of all MR and CT images from 2009 to 2010. CCRT: concomitant chemoradiation therapy, RT: radiotherapy, Cx: chemotherapy, and PDT: photodynamic therapy. This figure shows 1486 MR and CT studies performed in 1007 patients between 2009 and 2010. In 1068 cases no aberrations were found, and in 38 cases there were only benign tumors. In 25 cases encasement (>270 degrees encasement) was present at preoperative assessment. In 13 cases the report was not conclusive, and in 342 cases no encasement was seen. During operation in 2/125 = 1.6% cases, encasement of the internal carotid artery was found.

*(2) Preoperative Assessment of Encasement of the ICA.* CT- and MR image reports from 2009 to 2010 (*n* = 1486) were reviewed retrospectively for encasement of the ICA to estimate the prevalence of preoperatively diagnosed carotid encasement. After a first evaluation of the reports, 1106 out of the 1486 imaging reports were excluded (cases with no aberrations on imaging or with benign lesions were excluded; see Figure 2). Three hundred and eighty reports were evaluated for the presence of preoperative carotid encasement. These reports were from different radiologists using nonspecified criteria. Most of the radiologists used the criterion of >270 degrees circumferential involvement of the carotid artery as positive sign for encasement. However, it was unclear whether all radiologists used standardized criteria.

*(3) Evaluation of Radiologically Determined Criteria.* Twelve patients with peroperative encasement or preoperative encasement or possible encasement of the ICA were selected from the previously claimed cohorts. Their pretreatment MRIs (*n* = 6) and CTs (*n* = 6) were reviewed among 42 other scans (with no ICA encasement) by two experienced head and neck radiologists (JT and CL) using criteria selected from the literature [1, 2]. The observers were unaware of the peroperative findings, of all 54 scans. The results of only the 12 with ICA involvement were used for assessment of the interobserver variation. 

### 2.2. MR Technique

For this study both MRI examinations were performed at 1,5 T. (Magnetom; Siemens Medical Systems, Erlangen, Germany) and 3.0 T. (Philips Achieva release 3.2.1, Philips Medical Systems, Best, The Netherlands) using a dedicated 16-channel SENSE neurovascular coil. The following series were acquired: STIR TSE COR, TR (repetition time), IR (inversion time), TE (echo time) 3,880/180/20 ms, ETL: 12, FOV 300/228/40 mm, matrix: 320/320, 2 nex, slice thickness 4 mm; STIR TSE TRA, TR/IR/TE 4,228/180/20, ETL: 12, FOV: 180/200/80 mm, matrix 300/312, 2 nex, SW 3.5 mm, T1 TSE TRA, TR/TE: 780/10, ETL: 5, FOV 180/180/80, matrix 384/384, 2 nex, slice thickness: 3.5 mm; T1 3D Thrive (performed after intravenous injection of 15 cc gadoterate meglumine (Dotarem)), TR/TE: 5/2,22, ETL: 90, TA: 10, FOV 230/272/220, matrix 288/288, 2 nex, slice thickness: 0.8 mm; T1 TSE COR (postcontrast): TR/TE: 812/10, ETL: 6, FOV: 180/150/96 mm, matrix: 320/320, 3 nex, slice thickness 3.5 mm. 

The mean time between imaging and neck dissection was 12 days (range 1–48; SD 19).

### 2.3. CT Technique

CT studies were performed with one of two multidetector scanners (Philips Gemini TF or Siemens Sensation). Standard CT of the neck was performed, after the injection of nonionic contrast material (Omnipaque 300 mg/mL, GE Health Care, quantity in mL equal to body weight in kilograms) with an injection rate of 4 mL/sec. Acquisition of 1,5 or 2 mm slices started after 55 seconds, and the images were reformatted into 3-mm-thick sections in transverse and coronal directions.

### 2.4. Studied Radiological Criteria for ICA Encasement

Encasement of the ICA was assessed using the following radiological criteria selected from the literature [1, 2]:

1. encasement of the artery: none, 180–270, >270 degrees,
2. obliteration of the fat between the lymph node/primary tumor and the carotid artery,
3. deformation of the carotid artery,
4. length of contact between the carotid artery and tumor mass.

### 2.5. Statistics

Logistic regression was used to determine all significant characteristics for carotid encasement on MRI. To measure the interobserver agreement, the kappa coefficient was used. This coefficient can vary between −1 (complete disagreement) and +1 (complete agreement). If this measure takes on the value zero (0), the observer agreement can be interpreted as being the result of mere chance. A value of more than 0.75 can be interpreted as good agreement among observers. The overall kappa coefficient can be interpreted as a measure of agreement between the groups of observers. 

## 3. Results

### 3.1. Peroperative Assessment of Encasement of the ICA

In 24 of 260 cases (9.2%) peroperative encasement of both the internal or external carotid artery was found: in total 1.5% (4/260) of the cases undergoing a neck dissection had encasement of the ICA (see Figure 1). In one case of encasement of the ICA, clinical fixation of the tumor on physical examination was mentioned.

### 3.2. Preoperative Assessment of Encasement of the ICA

A total of 380 image reports were studied for the presence of preoperatively reported ICA encasement. In twenty-five cases (6.6%) the radiologist reported encasement. None of these patients were operated. In thirteen cases (3.4%) the radiologist reported possible encasement. Of these 13 patients, five underwent surgery and none had peroperative encasement. In 342 cases (90%) the radiologist reported no encasement. One hundred and twenty-five of these patients were operated; in two patients peroperative encasement of the ICA was present (2/125 = 1.6%), which was not reported during preoperative imaging (see Figure 2). 

### 3.3. Evaluation of Radiologically Determined Criteria

**Figure 3:**
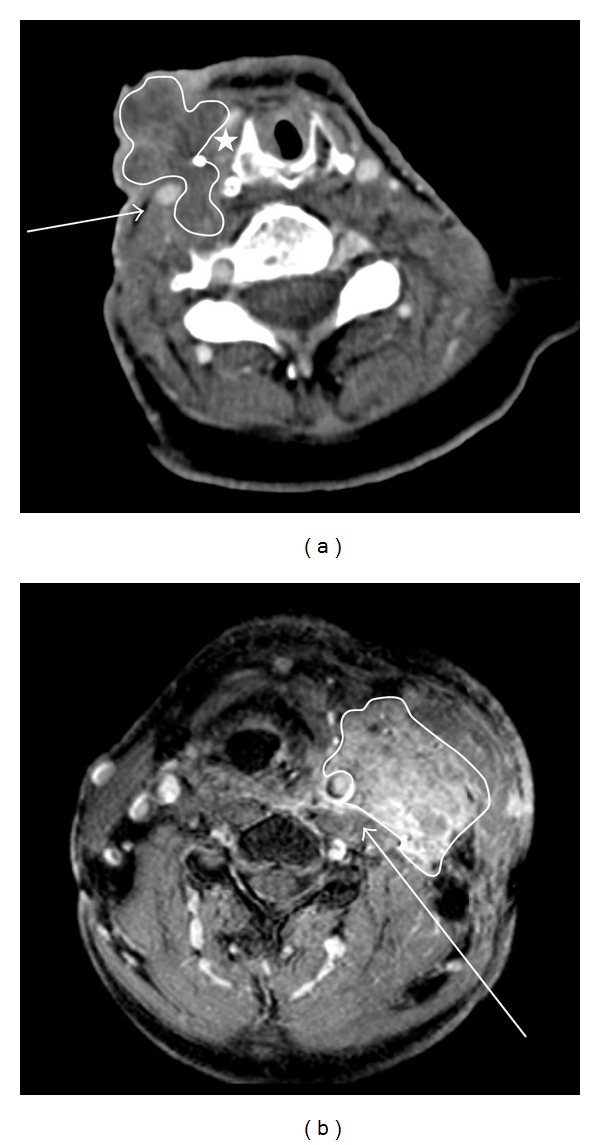
Examples of CT and MR images showing carotid encasement. (a) Axial CT image of a lymph node metastases (the mass is encircled by a white line) at the right side showing at least 270 degrees of encasement. The confluent lymph node mass is invading into the skin. The right carotid artery (arrow) is covered by the lymph node mass. Note: the right internal jugular vein is not visible, possibly due to compression. Suggestive the high-density structure (white star) lateral to the right lamina of the cricoid is surgical clip from earlier operation. (b) Fat-suppressed T1 contrast-enhanced MR section showing lymph node metastases in the left neck. The left internal carotid artery (arrow) is covered anteriorly and laterally by nodal disease (the mass is encircled by a white line). The circumferential involvement is (just) over 180 degrees.

Two radiologists reviewed 12 preoperative images of patients with known peroperative ICA encasement using the above-mentioned criteria (see Figure 3).  Table 1 shows the percentages of the radiologically determined criteria per observer and the interobserver variation. Interobserver kappa values were low with values from 0.273 (deformation of the carotid artery) to high with value of 1 (obliteration of fat planes) for the different parameters.

## 4. Discussion

### 4.1. Synopsis of Key/New Findings

These retrospectively studied cohorts demonstrate that preoperative assessment of encasement of the ICA using MRI and/or CT was missed in only 1.5%. However the criteria used in the literature show a high interobserver variation.

### 4.2. Comparisons with Other Studies

In 2010 Pons et al. [1] studied the relevance of five different imaging parameters for evaluating carotid artery invasion in 22 patients with peroperatively proven encasement of the ICA. Of these patients, preoperative CT and MR images were analyzed. Size of the adenopathy and intensity of the contact showed no correlation with peroperative findings. However, imaging characteristics such as carotid artery deformation, encasement of >180 degrees, and segmental obliteration of the fat were significantly associated (*P* < 0.05) with massive invasion of the carotid artery. In 1995 Yousem et al. [2] studied MR images of 53 carotid arteries in 49 patients. Twenty-two MR images had a tumor surrounding the carotid artery less than 180 degrees and none of these had carotid artery invasion at surgery. Seventeen arteries had more than 270 degrees of tumor encasement and twelve of these had invasion during surgery (12/17 = 71%). Fourteen arteries had tumor with 180–270 degrees of encasement on the preoperative imaging, with none having invasion at surgery. When the criterion of >270 degrees encasement was used, sensitivity of MRI was 100% and specificity 88%. In our series however, the criterion of 270 degrees resulted in an interobserver kappa value of 0.584.

Five articles reported on the value of preoperative CT imaging. Sarvanan et al. [5] studied 26 patients and compared palpation, ultrasound, and CT imaging. On CT, they studied encasement of >270 degrees and loss of fat planes. Sensitivity reached 75% and specificity 100%. Solano et al. [6] studied loss of a fat interface between the carotid and the neck mass. There were 11 false positive findings and one true positive finding. Rapoport et al. [7] studied in 2008 interobserver agreement based on a simplified two-item classification (0–50% and 51–100% involvement). The general kappa was 0.53. In our specific and selected series interobserver variation for categorical encasement (<180 versus 180–270 versus >270 degrees) was 0.584. Rothstein et al. [8] also studied loss of fat interface in 17 patients. All CT scans demonstrated this feature; however 16/17 = 94% was false positive. 

Yu et al. [9] studied in 2003 the diagnostic value of CT imaging for the detection of carotid encasement. In 27 patients, involvement of the common carotid artery or internal carotid artery (11 tumors) or the jugular vein (25 tumors) was studied. In 17 cases the tumors did not involve the cervical vessels. The compression and deformation, more than 180 degrees circumference, undefined carotid artery wall, and fat or fascial plane deletion between tumor and carotid wall were studied. With specificity ranging from 47.4% to 100% and sensitivity ranging from 18.5% to 90.9% they emphasized that a combination of criteria should be used. 

Our results seem to confirm the results from the above-mentioned studies. Overall, it can be questioned whether preoperative imaging assessment of carotid encasement for treatment selection should be used at all with no specific criteria available. 

The false negative rate of preoperative assessment of encasement of the ICA was 1.5% in our retrospective cohorts, using the intraoperative findings as “gold standard” for carotid encasement. If the radiologist reported >270 degrees of carotid encasement according to our current protocol, patients were not operated. For the calculation of observer variation we used a small selection of twelve patients. The interobserver kappa varied from 0.273 to 1.00 for the different radiologically determined characteristics. 

Various studies showed survival with carotid resection was less than 15 months [14, 15]. In a meta-analysis of Snyderman and D'Amico [16], 2-year disease-free survival was 22% after carotid resection. With these low survival figures in mind, one may seriously doubt whether carotid resection should be part of a standard surgical approach. 

### 4.3. Clinical Applicability of the Study

The importance of carotid artery encasement as a separate prognostic indicator justifying an aggressive surgical approach with a high risk of neurological complications can only be determined by a prospective multivariate analysis using standardized imaging techniques and agreement on radiological criteria. In daily practice we still have to rely on the limitations of preoperative imaging. Most probably the combination of head and neck surgical and radiological expertise remains of crucial importance to assess the resectability of neck node metastases in an individual patient. 

Future research efforts should be directed at more detailed depiction of the carotid artery wall. Increased resolution may give more insight in the amount of invasion of malignant neck disease in the various layers of the wall of the carotid artery. Use of high-field strength (3T) and application of surface coils may achieve this goal.

## 5. Conclusion

These retrospectively studied cohorts demonstrate that preoperative assessment of encasement of the ICA using MRI and/or CT was of value in evaluation of ICA encasement and therefore contributively in selecting operable patients (without ICA encasement), since in only 1.5% encasement was missed. However, observer variation affects the reliability of this feature. 

Most probably the combination of head and neck surgical and radiological expertise remains of crucial importance to assess the resectability of neck node metastases in an individual patient. The importance of carotid artery encasement as a separate prognostic indicator justifying an aggressive surgical approach with a high risk of neurological complications can only be determined by a prospective multivariate analysis using standardized imaging techniques and agreement on radiological criteria.

## Figures and Tables

**Table 1:** Radiologically determined criteria and interobserver kappa.

Radiologically determined criteria	Observer 1 *N* = 12	Observer 2 *N* = 12	Interobserver kappa
Encasement			0.584
<180 degrees	2 (17%)	0 (0%)	
180–270 degrees	0 (0%)	4 (33%)	
>270 degrees	10 (83%)	8 (67%)	
Obliteration of fat planes			1
No	0	0	
Yes	12 (100%)	12 (100%)	
Deformation of the carotid artery			0.273
No	4 (33%)	2 (17%)	
Yes	8 (67%)	10 (83%)	
Length of contact carotid artery			0.488
Mean in cm	3.5 (range: 1.0–5.0; SD 1.3)	3.6 (range: 1.6–6.1; SD 1.6)	
